# *Pogostemon cablin* extract as an anticancer agent on human acute myeloid leukemia

**DOI:** 10.1002/fsn3.2282

**Published:** 2021-05-02

**Authors:** Ju‐Huei Chien, Xiao‐Fan Huang, Wen‐Lin Lai, Kai‐Fu Chang, Chia‐Yu Li, Szu‐Yin Chen, Chun‐Yu Wu, Kuan‐Ying Li, Nu‐Man Tsai

**Affiliations:** ^1^ Department of Laboratory Medicine Taichung Tzu‐Chi Hospital Buddhist Tzu‐Chi Medical Foundation Taichung Taiwan, ROC; ^2^ Department of Medical Laboratory Science and Biotechnology Central Taiwan University of Science and Technology Taichung Taiwan, ROC; ^3^ Department of Medical Laboratory and Biotechnology Chung Shan Medical University Taichung Taiwan, ROC; ^4^ Institute of Medicine Chung Shan Medical University Taichung Taiwan, ROC; ^5^ Clinical Laboratory Chung Shan Medical University Hospital Taichung Taiwan, ROC; ^6^ Department of Life and Death Nanhua University Chiayi Taiwan, ROC; ^7^ Division of Cardiology Department of Internal Medicine Distmanson Medical Foundation Chia‐Yi Christian Hospital Chiayi Taiwan, ROC

**Keywords:** acute myeloid leukemia, anticancer, apoptosis, cell cycle, *Pogostemon cablin*

## Abstract

*Pogostemon cablin* has been indicated to treat many kinds of diseases and the progression of cancers, such as colorectal cancer. However, the effects of *P. cablin* extract (PPa extract) against acute myeloid leukemia have not been investigated. Thus, this study explored the anticancer potential of PPa extract and its mechanism in HL‐60 cells. The MTT assay results showed that PPa extract significantly inhibited the proliferation of HL‐60 cells in a dose‐dependent manner and affected cell morphology, causing cell shrinkage and the formation of debris. PPa extract blocked cell cycle progression at the G_0_/G_1_ phase in a dose‐ and time‐dependent manner and induced cell apoptosis, as shown by the observation of DNA fragments and apoptotic bodies. Furthermore, PPa extract caused the accumulation of a population of cells at G_0_/G_1_ phase via a reduction in p‐Rb, increasing p21 expression, and downregulating cell cycle regulator protein expression. Then, PPa extract was found to activate the extrinsic and intrinsic apoptosis pathways, leading to cell death. These data demonstrated that PPa extract exerted inhibitory activity and triggered cell apoptosis in HL‐60 cells and that PPa extract might be a chemopreventive agent for cancer therapy.

## Graphical abstract

Pogostemon cablin extract contained potential anticancer compound to suppress HL‐60 cell growth by blocking cell cycle progression and inducing cell apoptosis.

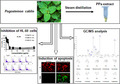

## 1 INTRODUCTION

Natural products have been strongly investigated as promising anticancer agents. Numerous studies suggest that vegetables and fruits play a protective role in reducing the risk of cancer (Tsuda et al., (2004); Gullett et al., 2010). *Pogostemon cablin* is a traditional herbal medicine used in the treatment of many kinds of diseases, such as the common cold, diarrhea, headache, and fever (Lin et al., 2014). Moreover, its other effects, including its anti‐inflammatory, antioxidant, antifungal, and antibacterial effects, have been widely reported (Kim et al., 2010, 2015; Su et al., 2012; Vu et al., 2016; Lu et al., 2011; Kocevski et al., 2013). *Pogostemon cablin* has been used as a complementary anticancer agent in colorectal cancer (Tsai et al., 2015). However, the mechanisms underlying the anticancer activity of *P. cablin* extract (PPa extract) in acute myeloid leukemia cells have yet to be understood. Acute myeloid leukemia (AML) is a hematological disease, and its incidence rate is 15%–20% in those who aged 15 years or younger and 80% in the patients above the age of 65 years (Deschler & Lübbert, 2006). The present chemotherapeutic approach for AML is the eradication of leukemia cells with a minimal reduction in normal cells, which has significantly improved the rate of remission. However, the relapse rate is more than 50%, with subsequent resistance leading to the death of most AML patients (Lagunas‐Rangel et al., 2017).

In recent years, the concept of cancer prevention has gained much attention, and the induction of apoptosis and cell cycle regulation have been suggested as targets in cancer treatment. Apoptosis, a process of programmed cell death in which cells are eliminated without the release of harmful substances, is essential for development and homeostasis. Moreover, it is activated by a serial caspase cascade including caspase‐8, caspase‐9, and caspase‐3, leading to cell apoptosis (Lopez & Tait, 2015; Koff et al., 2015; Baig et al., 2016; Goldar et al., 2015; Ichim & Tait, 2016). Cells progress through the cell cycle during cell proliferation, during which cell growth is controlled via cell cycle regulators and tumor suppressors, such as p53 and Rb (Jung et al., 2010; Giacinti & Giordano, 2006; Abbas & Dutta, 2009). Many studies have indicated that cancerous cells often present dysfunctional cell cycle regulation, causing uncontrolled cell proliferation. Thus, the purpose of this study was to investigate the antileukemic activity of PPa extract on apoptosis and cell cycle regulation in vitro.

## 2 MATERIALS AND METHODS

### 2.1 Cell lines and cell culture

HL‐60 (a human acute promyelocytic leukemia cell line), K562 (a human chronic myelogenous leukemia cell line), Jurkat (a human acute T‐cell leukemia cell line), P338D1 (a mouse macrophage‐like cell line), and RAW 264.7 (a mouse macrophage‐like cell line) cells were obtained from American Type Culture Collection. HL‐60, K562, P338D1, and RAW 264.7 cells were cultured in Dulbecco's modified Eagle's medium, and Jurkat cells were cultured in RPMI 1640 medium. The media were supplied with 10% fetal bovine serum, 1% penicillin/streptomycin, and 1% sodium pyruvate. Cells were incubated at 37°C in a humidified incubator containing 5% CO_2_. All other cell culture reagents were purchased from Gibco/Thermo Fisher Scientific, and all other chemicals were of research grade. HL‐60 and the FemtoPath TP53 exon 8 primer set was used to confirm the TP53 levels in K562 cells. The FemtoPath KRAS exon 2 primer set, FemtoPath EGFR exon 19 primer set, and FemtoPath PIK3CA exon 9 primer set (HongJing Biotech, New Taipei City, Taiwan) were used to confirm the EGFR, KRAS, and PIK3CA levels, respectively, in K562 cells.

### 2.2 Preparation of *Pogostemon cablin* extract

Fresh leaves of *Pogostemon cablin* plant from Indonesia were carried out with steam distillation and had been testified in our laboratory in small scale. Plant material (400 g) was penetrated with generated steam with 7.2 ml/min of flow rate at 100 ~ 105℃ for 100 min and had commissioned by Phoenix in large scale. PPa extract which was obtained from lipid layer was dissolved in DMSO to determine the concentration in μg/ml by the equation: 20 μl of PPa extract (g)/(180 μl of DMSO + 20 μl of PPa extract) (g). After that, the growth medium was utilized to dilute the PPa extract and the final concentration of DMSO in cells was less than 1%.

### 2.3 Cell viability assay

The effect of PPa extract on cell viability was determined by the MTS assay. The PPa extract was produced by steam distillation and obtained from PHOENIX. Cells (5 × 10^3^/50 μl) were grown in a 96‐well plate for 3–6 hr and then incubated with PPa at a series of concentrations for 12, 24, and 48 hr. After the treatment period, MTS was added to each well, and cells were further incubated for 12 hr. The absorbance at 490 nm was measured using a SpectraMax Plus 384 microplate reader (Molecular Devices). Cell viability is expressed as a percentage of the value for a control culture, which was considered 100% viable.

### 2.4 Flow cytometric analysis of the cell cycle

HL‐60 cells were plated at a density of 5 × 10^6^ cells/well in 10‐cm dishes in the presence of 0, 5, 15, and 25 μg/ml PPa extract for the indicated time intervals. The collected cells were stained with PI in the presence of RNase A and incubated at 4°C overnight. The DNA content was analyzed using a flow cytometer (BD) equipped with CellQuest Pro software.

### 2.5 TUNEL staining

HL‐60 cells were incubated with PPa extract (15 μg/ml) for 24 hr, and the cells were then gently smeared on slides to study apoptotic induction. The cells were fixed with 10% formalin in the presence of methanol, followed by detection with an in situ cell death detection kit (Roche, Germany) according to the manufacturer's instructions. After incubation, the slides were washed and immediately visualized under a fluorescence microscope (ZEISS Axioskop2, USA) at 400× magnification to detect apoptotic cells.

### 2.6 Total protein extraction and Western blot analysis

HL‐60 cells were treated with PPa extract (0, 5, 15, and 25 μg/ml) for 6, 12, 24, and 48 hr. To extract the total protein, the collected cells were lysed with lysis buffer. Cell lysates were separated by SDS‐PAGE, and proteins were transferred to PVDF membranes. After membrane blocking, the membranes were incubated with primary antibodies at 4°C overnight, followed by incubation with HRP‐conjugated secondary antibodies. Subsequently, the blots were detected using the T‐Pro LumiFast Plus chemiluminescence detection kit (T‐Pro Biotechnology, USA), and signals were captured using an Image Quant LAS 4000 image reader (GE Healthcare Life Sciences). Primary antibodies against p‐Rb, p21, PCNA, cdk2, cdk4, cyclin B1, cyclin D1, FAS, bax, VEGF, MMP2, MMP9, caspase‐3, caspase‐8, and caspase‐9, and horseradish peroxidase (HPR)‐conjugated secondary antibody were purchased from Santa Cruz Biotechnology, Inc. and iReal Biotechnology Co., Ltd. These data were performed three independently experiments.

### 2.7 GC/MS analysis

Gas chromatography–mass spectrometry (GC‐MS) analyses of the sample were performed using an Agilent 7890CB gas chromatograph (AccuTOF‐GCx) with an Rxi‐5MS capillary column (film thickness: 30 m × 0.25 mm ×0.25 μm) that was commissioned to the National Central Taiwan University Office of Research and Development's Center for Advanced Instrumentation. The sample was diluted using hexane (1/500). The oven temperature was programmed to 300⁰C/1 ml/min. Helium was used as carrier gas at flow 1.0 ml/min. The identification of compounds was based on comparison of their mass spectra with those of WILEY and NIST Libraries.

### 2.8 Statistical analysis

The results are presented as the mean ± standard deviation (*SD*). Statistical analyses between various groups were performed by Student's *t* test. Significance was indicated by *p* < .05 in all experiments.

## 3 RESULTS

### 3.1 PPa extract inhibited the growth of leukemia cells

**FIGURE 1:**
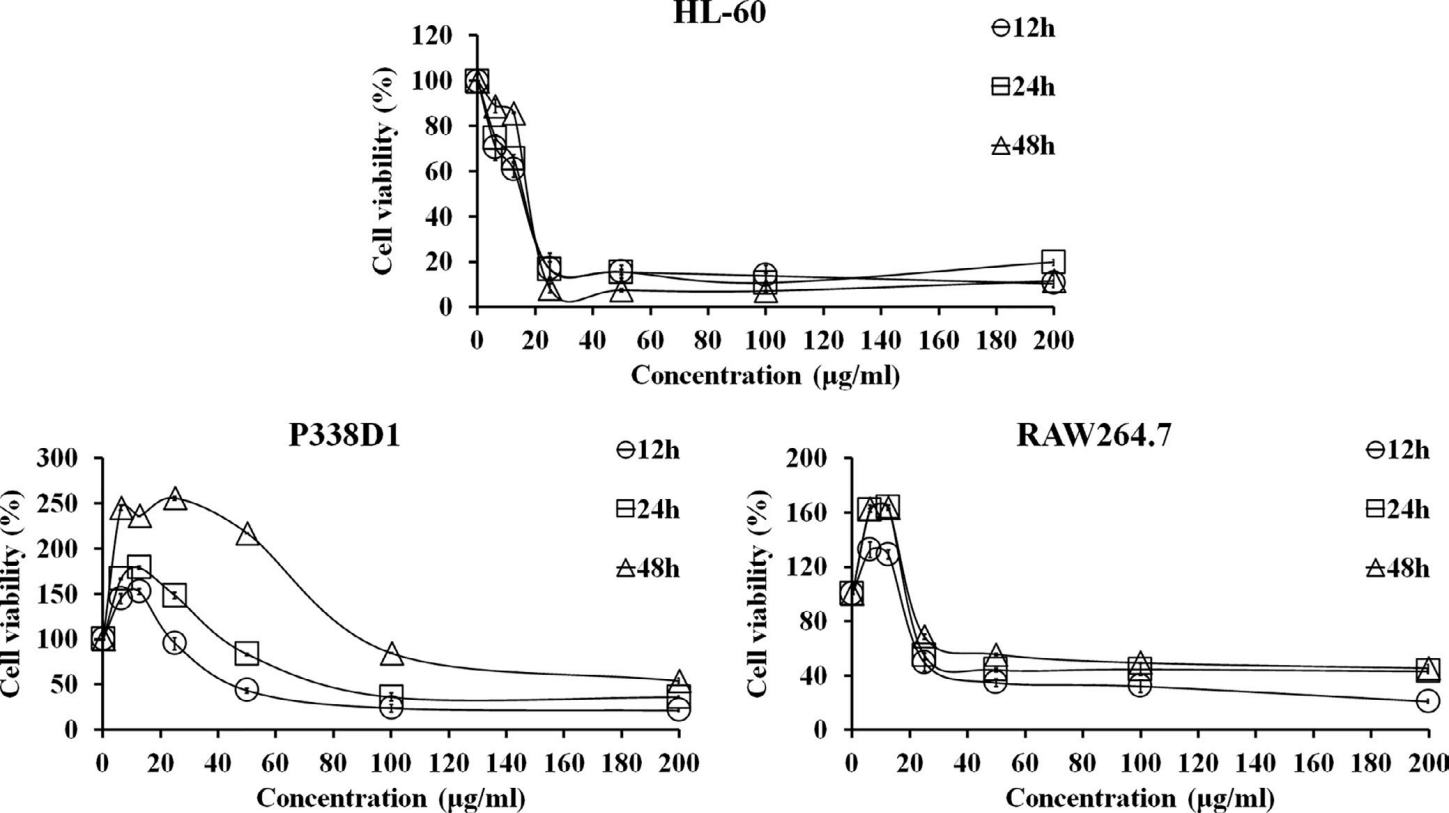
Inhibitory effects of PPa extract on cell proliferation in HL‐60, RAW 264.7, and P338D1 cells. Cells were treated with PPa extract at different concentrations (0–200 μg/ml) for 12, 24, and 48 hr, and cell viability was then assessed by MTS assay. The results are presented as the mean ± *SD* of three independent experiments

The inhibitory effect of PPa extract against HL‐60, RAW 264.7, and P338D1 cells was measured by MTS assay. The cells were treated with PPa extract at a series of concentrations for 12, 24, and 48 hr, as shown in Figure 1. PPa extract significantly inhibited the growth of these cells in a dose‐dependent manner. For HL‐60 cells, treatment with PPa extract at a lower concentration (<25 μg/ml) sharply decreased cell viability, and treatment with PPa at concentrations ranging from 50 to 200 μg/ml had a marked inhibitory effect. Furthermore, HL‐60 cells were more sensitive to PPa extract treatment for 48 hr compared to other periods. However, treatment with PPa extract at a low concentration (<25 μg/ml) for 12, 24, and 48 hr significantly increased the proliferation of RAW 264.7 and P338D1 cells. As a result, PPa extract showed the ability to induce normal macrophage proliferation instead of an inhibitory effect on normal macrophages; in contrast, PPa extract had a highly efficient inhibitory effect on HL‐60 cells. To further examine the inhibitory effect of PPa extract on the proliferation of different types of leukemia cells, Jurkat (Acute lymphocytic leukemia, ALL) and K562 (Chronic myeloid leukemia, CML) cells were also investigated by MTS assay, as shown in Table 1. The IC_50_ of PPa extract at 12 and 24 hr was 15.58 ± 0.45 and 16.43 ± 0.11 μg/ml in HL‐60 cells; 16 ± 2.69 and 17.56 ± 3.04 μg/ml in Jurkat cells; and 21.78 ± 1.39 and 34.89 ± 0.46 μg/ml in K562 cells, respectively. PPa extract treatment had a higher inhibitory effect on HL‐60 and Jurkat cells than on K562 cells. PPa extract exhibited IC_50_ values ranging from 24 to 85 μg/ml in normal macrophages, suggesting that PPa extract shows low cytotoxic activity against normal macrophages. These results revealed that PPa extract exerts a stronger inhibitory effect on leukemia cells in a dose‐dependent manner and lower cytotoxicity against normal macrophages; moreover, PPa extract showed the ability to induce macrophage proliferation.

**TABLE 1:** IC_50_ of PPa extract in leukemia and normal cell lines

Cell line	Cell type	PPa extract (treatment time and IC_50_; μg/ml)	
**Leukemia cell lines**			
HL‐60	Human AML	12 hr	15.58 ± 0.45^a^
		24 hr	16.43 ± 0.11^a^
Jurkat	Human ALL	12 hr	16.00 ± 2.69^a^
		24 hr	17.56 ± 3.04^a^
K562	Human CML	12 hr	21.78 ± 1.39^a^
		24 hr	34.89 ± 0.46^a^
**Normal cell lines**			
RAW 264.7	Mouse macrophage	12 hr	24.95 ± 0.13
		24 hr	36.30 ± 0.60
P338D1	Mouse macrophage	12 hr	46.83 ± 0.4
		24 hr	85.36 ± 0.5

Note

values are mean ± *SD*. The values represented at least three independent experiments. a: It was significant different that compared with tumor cells and normal cells, *p* < .05.

Abbreviations: ALL, acute lymphoblastic leukemia; AML, acute myeloid leukemia; CML, chronic myelogenous leukemia.

### 3.2 PPa extract‐induced cell cycle arrest in HL‐60 cells

**FIGURE 2:**
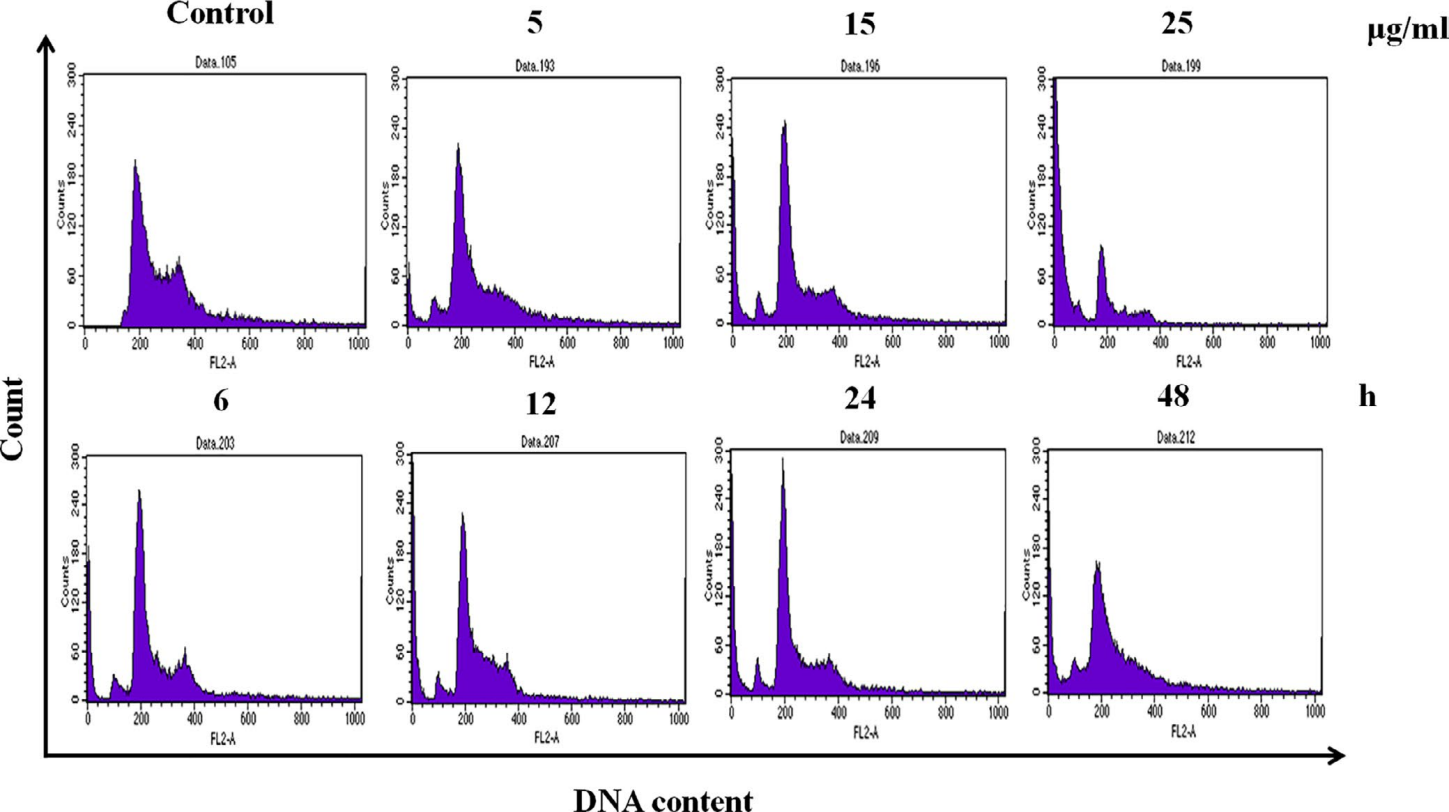
Effects of PPa extract on HL‐60 cell cycle distribution. HL‐60 cells were treated with PPa extract (5, 15, and 25 μg/ml) for the indicated duration, and the collected cells were stained with propidium iodide (PI) and analyzed for their DNA content by flow cytometry

To elucidate the mechanisms underlying the inhibitory effect of PPa extract on cell growth, the cell cycle distribution of HL‐60 cells was analyzed by flow cytometry. HL‐60 cells were treated with PPa extract (5, 15, and 25 μg/ml) for 24 hr, and the population of cells in G_0_/G_1_ phase significantly increased from 48.3% to 68.1%, accompanied by a decrease in the population of cells in S phase from 13.8% to 4.4% and in the population of cells in G_2_/M phase from 31.4% to 11.6%. Similarly, after PPa extract treatment (15 μg/ml) for 6, 12, 24, and 48 hr, the accumulation of cells in G_0_/G_1_ phase was markedly increased, followed by a decrease in the percentage of cells in the S and G_2_/M phases, as shown in Table 2. These results indicate that PPa extract can induce cell cycle arrest at G_0_/G_1_ phase in a dose‐ and time‐dependent manner, as shown in Figure 2. Furthermore, these results suggest that the inhibitory effect of PPa extract on HL‐60 cell is due to the induction of cell cycle arrest at G_0_/G_1_ phase.

**TABLE 2:** PPa extract induced the arrest of HL‐60 cells in the G_0_/G_1_ phase of the cell cycle

Treatment	Percentage of cells (%)		
	G_0_/G_1_	S	G_2_/M
Control	48.32 ± 0.62*	13.82 ± 1.23^#^	31.38 ± 2.02^#^
PPa (5 μg/ml)	65.06 ± 1.67*	13.73 ± 0.45^#^	18.09 ± 0.00^#^
PPa (15 μg/ml)	66.27 ± 0.22*	11.45 ± 0.17^#^	18.81 ± 0.14^#^
PPa (25 μg/ml)	68.13 ± 1.44*	4.38 ± 0.46^#^	11.56 ± 0.72^#^
PPa (6 hr)	71.17 ± 0.24*	7.74 ± 0.09^#^	18.76 ± 0.28^#^
PPa (12 hr)	67.64 ± 0.69*	12.23 ± 0.85^#^	15.47 ± 1.04^#^
PPa (24 hr)	71.42 ± 0.64*	9.21 ± 0.21^#^	15.67 ± 0.33^#^
PPa (48 hr)	73.81 ± 1.75*	9.40 ± 0.19^#^	12.43 ± 1.38^#^

Note

Values are the mean ± *SD*. Significantly increased (*) or decreased (#) compared with the control treatment (*p* < .05). The values from three independent experiments were analyzed.

### 3.3 PPa extract‐induced apoptosis in HL‐60 cells

**FIGURE 3:**
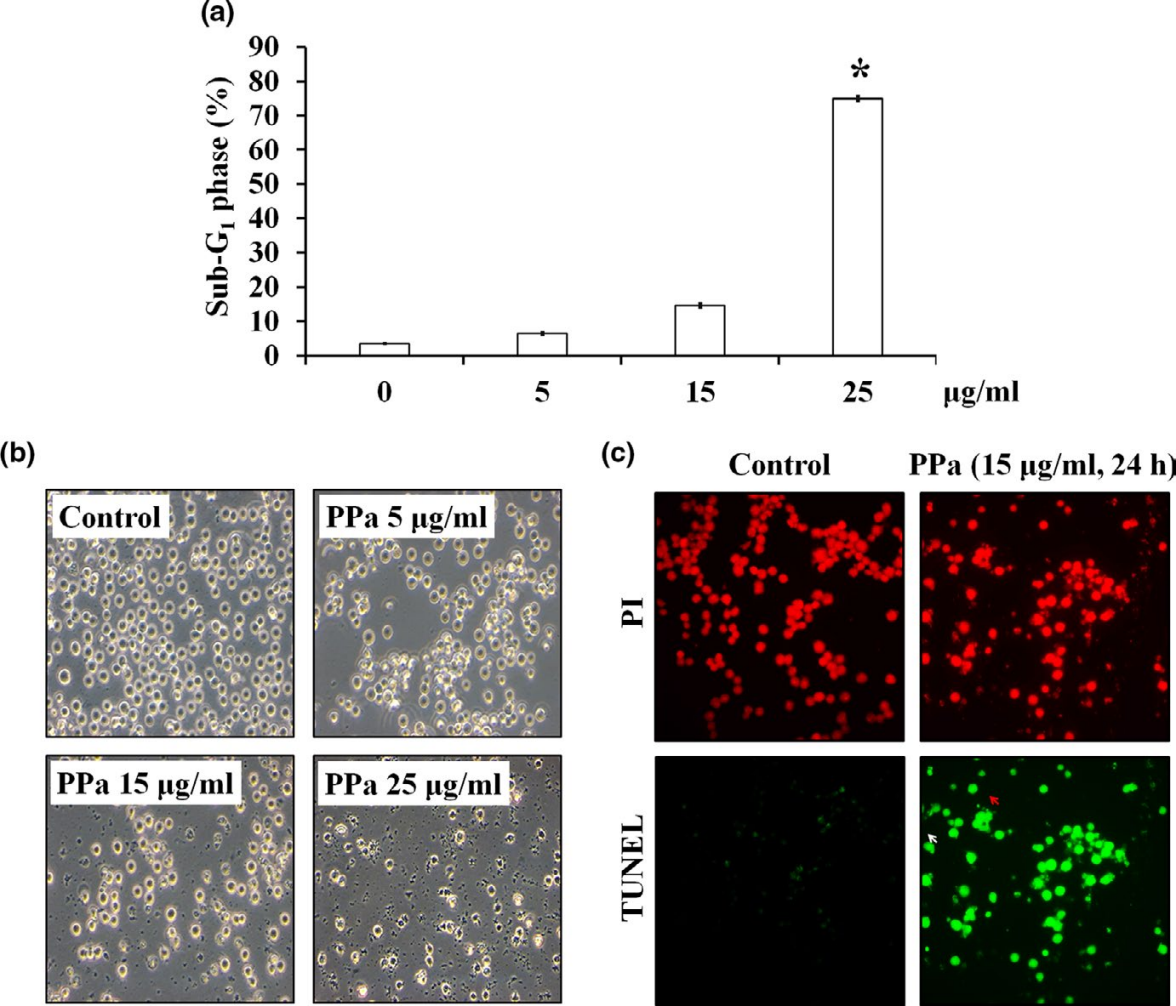
PPa extract induced apoptosis in HL‐60 cells. (a) HL‐60 cells were treated with PPa extract at different concentrations (0, 5, 15, and 25 μg/ml), and after 24 hr of treatment, morphological changes were investigated by microscopy. (b) The percentage of cells in the sub‐G_1_ phase after PPa extract exposure (0, 5, 15, and 25 μg/ml) for 24 hr was calculated. (c) HL‐60 cells were treated with PPa extract (15 μg/ml) for 24 hr. Morphological changes were determined by fluorescence microscopy. DNA fragments (red arrow) and apoptotic bodies (white arrow) are indicated. All experiments were independently performed three times, and representative results are shown. Statistically significant differences are indicated with *p < .05

The results of cell cycle distribution analysis also revealed that the percentage of cells in the sub‐G_1_ phase was increased after PPa extract treatment in a dose‐dependent manner (Figure 3a). Moreover, microscopic examination revealed that HL‐60 cells exposed to PPa extract (5, 15, and 25 μg/ml) exhibited cell swelling, shrinking, and debris, indicating that PPa extract contributed to HL‐60 cell death (Figure 3b). To further detect the ability of PPa to induce apoptosis, HL‐60 cells were incubated with PPa extract (15 μg/ml) for 24 hr and subjected to a TUNEL assay, followed by PI staining. As shown in Figure 3c, HL‐60 cells without PPa extract treatment contained intact and round nuclei, and nearly no TUNEL‐positive cells (green) were observed. In contrast, after PPa extract treatment, HL‐60 cells presented an increased proportion of apoptotic cells, culminating in DNA fragmentation and apoptotic body formation (Figure 3c). These results demonstrated that PPa extract induced cell apoptosis in HL‐60 cells.

### 3.4 Effects of PPa extract on the cell cycle and cell apoptosis

**FIGURE 4:**
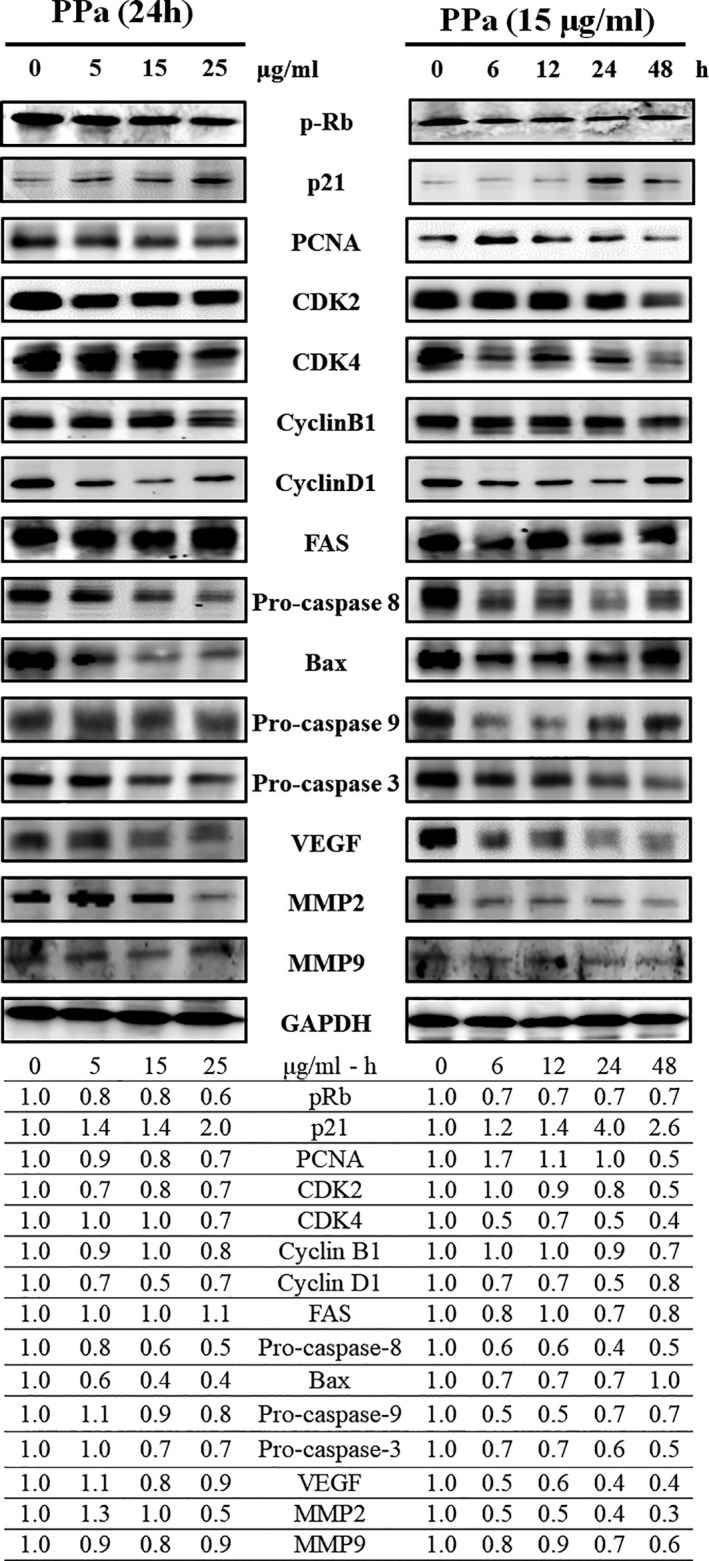
PPa extract induced cell cycle regulation and apoptosis activation. HL‐60 cells were treated with PPa extract at different concentrations for the indicated periods. Protein expression with or without PPa extract treatment was determined by Western blot analysis (GAPDH was used as a loading control.). The experiment was repeated three times, and representative blots are shown

**FIGURE 5:**
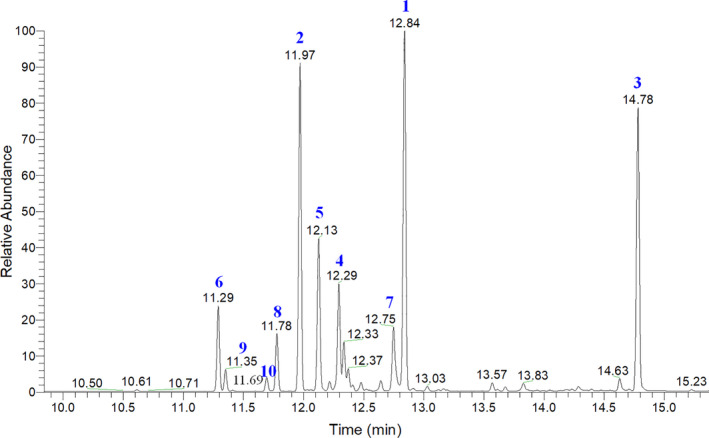
GC/MS analysis of PPa extract. 1: α‐Guaiene; 2:α‐Selinene; 3: Patchouli alcohol; 4: α‐Patchoulene; 5: Seychellene; 6: α‐Gurjunene; 7: Aciphyllene; 8: Caryophyllene; 9: α‐Elemene; 10: Naphthalene

To further clarify the molecular mechanisms underlying the inhibitory effects of PPa extract on HL‐60 cell growth, we examined the effects of PPa extract on key protein markers associated with the cell cycle and apoptosis. Rb is a tumor‐suppressor protein that is inactivated upon its phosphorylation, allowing cell cycle progression. p21 is a cyclin‐dependent kinase inhibitor results in decreased cell proliferation. Immunoblot analysis indicated that PPa extract downregulated the expression of p‐Rb, whereas the expression of p21 was upregulated in a drug concentration manner with PPa extract treatment. Then, the expression of the downstream cell cycle regulators PCNA, cdk2, cdk4, cyclin B1, and cyclin D1 was shown to be decreased as well (Figure 4). To determine whether this apoptotic expression pattern was involved in PPa extract‐induced apoptosis in HL‐60 cells, extrinsic and intrinsic apoptosis‐related proteins were investigated by Western blot analysis. As shown in Figure 4, the expression of FAS increased, while that of procaspase 8 decreased, suggesting that PPa extract activated the extrinsic apoptotic pathway. The expression of bax, a key intrinsic mitochondria‐mediated apoptotic protein, increased, while the expression of procaspase 9 decreased, contributing to the PPa‐induced intrinsic apoptotic pathway. Caspase‐3 plays an important role in both death receptor‐ and mitochondria‐mediated apoptosis. The expression of procaspase 3 was decreased after PPa extract treatment, revealing that PPa extract induced both the extrinsic and intrinsic apoptotic pathways, resulting in cell apoptosis. These findings demonstrated that PPa extract induced cell cycle arrest through p21 activation and cell apoptosis via activation of the extrinsic and intrinsic apoptotic pathways. Subsequently, to further analyzed the antileukemia components in PPa extract, GC/MS analysis was performed and the data were shown as Figure 5. Table 3 revealed that the ten major components in PPa extract were azulene (21.81%), α‐guaiene (18.85%), patchouli alcohol (18.16%), α‐patchoulene (11.14%), seychellene (9.31%), α‐gurjunene (4.96%), α‐selinene (4.80%), caryophyllene (3.38%), cyclohexane (1.32%), and naphthalene (0.89%), and the structure of those compound were shown in Table 4.

**TABLE 3:** GC/MS quantification of dominant compounds in PPa extract

Number	RT	Compound	Percentage (%)	CAS number
1	12.84	Azulene	21.81%	22567‐17‐5
2	11.97	α‐Guaiene	18.85%	3691‐12‐1
3	14.78	Patchouli alcohol	18.16%	5986‐55‐0
4	12.29	α‐Patchoulene	11.14%	560‐32‐7
5	12.13	Seychellene	9.31%	20085‐93‐2
6	11.29	α‐Gurjunene	4.96%	489‐40‐7
7	12.75	α‐Selinene	4.80%	473‐13‐2
8	11.78	β‐Caryophyllene	3.38%	87‐44‐5
9	11.35	Cyclohexane	1.32%	515‐13‐9
10	11.69	Naphthalene	0.89%	4630‐07‐3

Abbreviation: RT, Retention time.

**TABLE 4:**
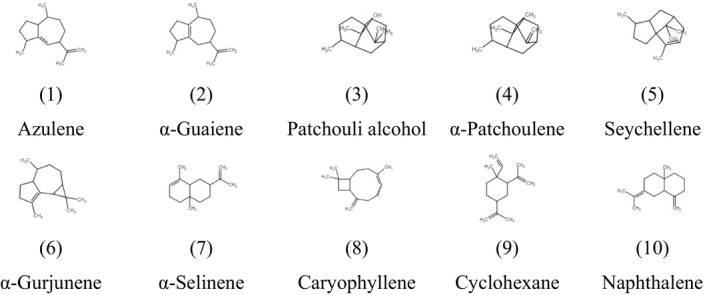
Structures of isolated compound from PPa extract

## 4 DISCUSSION

Natural plants have been widely used to treat many kinds of diseases for centuries and are also used as a daily medicine or functional food for the prevention of disease. With the incidence of cancer, preventative medicine has received more attention worldwide and has been rapidly expanding, even in developed countries, in recent years. For instance, traditional herbal medicine accounts for 30%–50% of the total medicinal consumption in China (Tsuda et al., 2004; Gullett et al., 2010). *P. cablin* is an herbal plant that has been used widely to treat fever and inflammation and to alleviate pain (Lin et al., 2014). In this study, *P. cablin* extract exerted an antiproliferative effect and inhibited the growth of three types of leukemia cells, HL‐60, Jurkat, and K562 cells. Among these cell lines, HL‐60 and Jurkat cells were more sensitive to PPa extract treatment, while K562 cells exhibited high tolerance to PPa extract due to drug resistance. Furthermore, PPa extract at a low dose induced macrophage proliferation and had a less cytotoxic effect on macrophages, suggesting that PPa extract displays few side effects.

Cancer chemotherapeutic agents may alter cell cycle regulation, resulting in cellular arrest; hence, diminishing cell proliferation and inducing apoptosis in cancerous cells have been common mechanisms in cancer therapy. Next, we further explored the inhibitory effects of PPa extract on HL‐60 cells. PPa extract induced marked cell cycle arrest at G_0_/G_1_ phase, followed by a decline in the percentage of cells at S and G_2_/M phase. In addition, the effect of PPa extract on the Rb protein, a hallmark of the G_1_ to S transition during the cell cycle, and p21WAF1, which has been characterized as a tumor suppressor due to its role in mediating cell cycle arrest, has been examined (Jung et al., 2010; Giacinti & Giordano, 2006; Abbas & Dutta, 2009). Cell cycle arrest at G_0_/G_1_ phase with decreases in p‐Rb protein and p21 levels imply that the expression of cyclin and cyclin‐dependent kinase was reduced by PPa extract in HL‐60 cells. Apoptosis induction is a tool used for the management of chemotherapy. In the present study, a TUNEL assay indicated that PPa extract treatment obviously induced the generation of DNA fragments and apoptotic bodies, which are direct indicators of cellular apoptosis. Apoptotic activation primarily occurs via mitochondria‐ or death receptor‐dependent signaling cascades. Procaspase‐8 is cleaved by binding the FADD complex, and procaspase‐9 binds Apaf1, leading to the activation of caspase‐9. Subsequently, downstream effector caspases are activated, finally triggering apoptosis. The results of the present study indicated that PPa extract treatment significantly activated both mitochondria‐ and death receptor‐dependent apoptosis pathways. Taken together, our data strongly suggest that the PPa extract‐mediated inhibition of HL‐60 cell growth is the consequence of cell cycle arrest during the G_0_/G_1_ transition and the induction of cell apoptosis. PPa‐mediated inhibition of cell proliferation and induction of apoptosis in leukemia cells provide new insight into the chemopreventive effects of PPa extract. Furthermore, GC/MS analysis indicated the ten main compounds in PPa extract and, among these, azulene, patchouli alcohol, and β‐caryophyllene have been reported the anticancer activity. Azulene has been reported the inhibitory ability on breast cancer cells (MCF‐7) and prostate cancer cells (DU145) (Ayaz et al., 2020). Patchouli alcohol exerts anticancer activity on colon and lung cancer (Lu et al., 2016; Jeong et al., 2013). β‐caryophyllene presents antimelanoma ability on B16F10 melanoma cells in high‐fat diet‐induced obese C57BL/6N mice (Jung et al., 2015; Amiel et al., 2012).

In conclusion, *P. cablin* extract induced cell cycle arrest and apoptosis, which might have become new therapeutic targets in human myeloid leukemia cells for cancer treatment. These results provide new insights into the possible molecular machinery underlying the activity of PPa extract, indicating its potential as a promising chemopreventive agent for cancer treatment or as a daily preventive medicine. However, further investigation of the components of PPa extract responsible for its efficacy will be important for the application of PPa extract.

## CONFLICT OF INTEREST

The authors declare that they have no conflict of interest.

## Data Availability

The data that support the findings of this study are available from the corresponding author upon reasonable request.
